# Minimally invasive biopsy‐based diagnostics in support of precision cancer medicine

**DOI:** 10.1002/1878-0261.13640

**Published:** 2024-03-22

**Authors:** Bo Franzén, Gert Auer, Rolf Lewensohn

**Affiliations:** ^1^ Department of Oncology‐Pathology Karolinska Institutet Stockholm Sweden; ^2^ Cancer Centre Karolinska (CCK) Foundation Karolinska University Hospital Stockholm Sweden; ^3^ Theme Cancer, Medical Unit Head and Neck, Lung, and Skin Tumors, Thoracic Oncology Center Karolinska University Hospital Stockholm Sweden

**Keywords:** biomarkers, fine‐needle aspirates, immune signaling, minimally invasive diagnostics, solid tumors, tumor microenvironment

## Abstract

Precision cancer medicine (PCM) to support the treatment of solid tumors requires minimally invasive diagnostics. Here, we describe the development of fine‐needle aspiration biopsy‐based (FNA) molecular cytology which will be increasingly important in diagnostics and adaptive treatment. We provide support for FNA‐based molecular cytology having a significant potential to replace core needle biopsy (CNB) as a patient‐friendly potent technique for tumor sampling for various tumor types. This is not only because CNB is a more traumatic procedure and may be associated with more complications compared to FNA‐based sampling, but also due to the recently developed molecular methods used with FNA. Recent studies show that image‐guided FNA in combination with ultrasensitive molecular methods also offers opportunities for characterization of the tumor microenvironment which can aid therapeutic decisions. Here we provide arguments for an increased implementation of molecular FNA‐based sampling as a patient‐friendly diagnostic method, which may, due to its repeatability, facilitate regular sampling that is needed during different treatment lines, to provide tumor information, supporting treatment decisions, shortening lead times in healthcare, and benefit healthcare economics.

## Graphical abstract

Fine‐needle tumor aspiration (FNA) permits molecular analyses of tumors including mutations, RNA transcripts and proteins. It lowers the risk of complications related to tumor sampling compared with core needle biopsies at diagnosis. FNA which is patient‐friendly more readily permits re‐biopsies at times of treatment decision making i.e., partial remissions, emerging treatment resistance at different lines of various treatments. Created with BioRender.com.

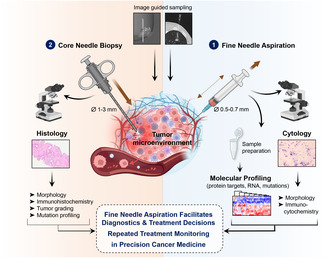

**Abbreviations**
ALDH1: aldehyde dehydrogenase 1 family member A1
BC: breast cancer
BM: bio‐marker
BRAF: proto‐oncogene B‐Raf
CCL2: CCL3, CCL4, CCL23, C‐C motif chemokine ligands 2, 3, 4 and 23
CIN: chromosomal instability
CNB: core needle biopsy
CT: computed tomography
EBUS‐FNA: endoscopic bronchoscopy ultrasound‐guided FNA
EML4‐ALK: echinoderm microtubule‐associated protein‐like 4 – anaplastic lymphoma kinase fusion gene
EMT: epithelial‐to‐mesenchymal transition
EpCAM: epithelial cell adhesion molecule
ER: estrogen receptor
EUS‐FNA: endoscopic ultrasound‐guided FNA
EZR‐ROS1: Ezrin – ROS proto‐oncogene 1 (receptor tyrosine kinase) fusion gene
FNA: fine‐needle aspiration biopsy
HER‐2: human epidermal growth factor receptor 2
HNSCC: head and neck squamous cell carcinoma
ICC: immuno cytochemistry
IL‐6: IL‐8, IL‐12, interleukins 6, 8 and 12
MET: MET proto‐oncogene, receptor tyrosine kinase
MNB: medium needle biopsy
MR or MRI: magnetic resonance imaging
NGS: next‐generation sequencing
NK Cell: natural killer cell
NSAIDs: non‐steroidal anti‐inflammatory drugs
NSCLC: non‐small cell lung cancer
PaC: pancreatic cancer
PC: prostate cancer
PCM: precision cancer medicine
PD‐L1: programmed death‐ligand 1
PTC: papillary thyroid cancer
RAS: rat sarcoma virus oncogene
RET: rearranged during transfection proto‐oncogene
ROSE: rapid on‐site evaluation
SGC: salivary gland cancers
TERT: telomerase reverse transcriptase
TGF‐β1: tissue growth factor beta 1
TME: tumor microenvironment
TRUS: transrectal ultrasound
TTF1: transcription termination factor 1
TTNB: transthoracic needle biopsy
US: ultrasound

## 1 Introduction

The continuing global increase in cancer incidence as well as the cancer prevalence results in increased healthcare costs related to both initial diagnostic testing, and to repeated testing during disease progression. In 2020, around 4 million new cancer cases were diagnosed in Europe, and 1.9 million people died due to cancer [1]. Furthermore, incidence is estimated to increase by 21% by 2040 [European Cancer Information System, 16^th^ March 2022, https://ecis.jrc.ec.europa.eu/], while prevalence is expected to increase in parallel, reaching 13.5 million cases in Europe by 2025 (Globocan, IARC, and WHO). At the same time, the introduction of novel and precise treatments, stimulates a growing demand for patient‐friendly, repeatable diagnostics at various stages of treatment to guide therapeutic interventions. At the same time, there is a need for advanced molecular information from representative and repeated tumor samples to support the development and evaluation of new therapies and to address therapeutic resistance.

In recent decades, the gold standard for tumor sampling has been based on CNB or medium needle biopsy (MNB), that provide tissue samples for histopathology and immunohistochemistry (IHC) based examinations. While techniques to acquire molecular information such as targeted mutation analysis and spatial omics from CNB samples have been developed, the CNB sampling procedure *per se* may increase the risk of adverse effects, such as. inflammation, infection, sepsis (transrectal prostate cancer biopsies) and pneumothorax (trans thoracic lung cancer biopsies). Moreover, extensive longitudinal repetitions of CNB sampling for therapy monitoring is not always feasible [2]. In breast cancer (BC) diagnostics, other concerns with CNB have also been raised [3]. An alternative method; fine‐needle aspiration (FNA) biopsy and cytopathology (or interventional cytology), that was developed at Karolinska more than five decades ago [4] offers a number of benefits; (a) a patient‐friendly minimally traumatic sampling procedure, usually with no need for local anesthesia, (b) a minimal risk of sampling‐related adverse effects, (c) imaging [computed tomography (CT), magnetic resonance (MR) or ultrasound (US)]‐guided sampling of small, hard‐to‐reach lesions at multiple locations (which may reflect tumor heterogeneity) can be taken, at a single sampling session, (d) repeated sampling; at initial diagnosis, monitoring during the course of therapy, and sampling of residual disease at resistance to guide alternative treatments, (e) rapid sample processing for preliminary diagnostics by cytology and verification of sample representativity. Recent studies show that FNA sampling provides higher tumor cellularity and higher tumor fractions than CNB sampling [5]. However, the minimal amount of material obtained by FNA [*i.e*., microliters of cell suspension from the tumor microenvironment (TME)] have previously limited the information that can be gathered, and the technique has also been hampered by a lack of expertise in cytopathology. As a result, the request for FNA‐based routine diagnostics decreased during the 1990s while CNB‐based diagnostics increased. An additional aspect was the need to visualize the invasive tumor front and morphological context, which is core of histopathology and CNB‐based cancer diagnostics. However, it is likely that molecular signatures could be identified in the future that can be used to assess invasive capacity in FNA samples. Technologies for genomic (i.e. multiplex mutation and gene alteration profiling) and targeted proteomics (i.e. multiplex protein and/or protein target profiling) analysis of solid cancers via molecular panels are in rapid development and could potentially be applied to both CNB and FNA samples [6]. This article aims to review possible complications associated with CNB and, in parallel, describe recent advances in FNA‐based molecular cytopathology (which may offer unprecedented molecular diagnostics based on molecular characteristics) for treatment decisions. With this in mind, we also discuss potential opportunities to gradually replace CNB sampling with molecular cytology‐based FNA sampling when clinical validation studies support this transition and hope to stimulate a constructive discussion on the impact of tumor sampling methods in the emerging era of molecular‐based precision cancer medicine.

## 2 Core needle biopsy‐associated complications

### 2.1 Background

As early as 1990, Nishizaki et al. [7] showed that traumatic manipulation of VX2 carcinoma in rabbits enhanced metastasis. In a clinical context, complications from prostate CNB have been studied in more detail and up to 25% lower urinary tract complications have been reported [8]. The advantages and disadvantages regarding CNB have been debated for decades and recent studies do not indicate that this debate is over. For instance, Joosse et al. recently showed that a biopsy‐related (transrectal ultrasound/TRUS‐guided CNB) increase of circulating tumor cells (CTCs) in prostate cancer was significantly correlated with a worse progression‐free survival independent of Gleason score [9, 10].

Biopsy of suspicious lesions is still essential for diagnostics. However, the ability to efficiently and safely provide biopsy samples for diagnostic and prognostic analysis remains limited with regard to current diagnostic requirements [11]. In this review we argue that needle biopsy adequacy can be *improved substantially* by; optimizing needle biopsy procedural techniques, implementing modern molecular analytic methods, and improving clinical practice and education—all of which can contribute to increased benefits for cancer patients and to better health economics.

Most studies that compare CNB and FNA focus on diagnostic accuracy. In some cases, such as in the diagnosis of thyroid nodules, FNA is the primary choice [12]. In others, such as in the diagnosis of breast lesions, CNB has been the preferred method over the last two decades due to higher accuracy [13, 14].

Clinical studies comparing FNA with CNB have so far shown that representative FNA material is collected with similar diagnostic accuracy. However, single cells and isolated cell complexes obtained by FNA are more difficult to interpret by microscopic examination compared with histological material and require longer histopathology/laboratory experience. An increasing number of studies indicate that this drawback may be resolved by ancillary methods, such as molecular testing, as is described in the second part of this review.

In this context, it is noteworthy that only a few studies have been designed to *exclude* the possibility that CNB (compared with FNA) increases the risk of dissemination or progression in subtypes of cancers that are highly malignant, usually aneuploid and characterized by chromosomal instability (CIN) [15, 16]. Here, the recommendation is that diagnostic CNB biopsies should be avoided in cancers such as primary malignant melanoma and ovarian cancers without signs of metastasis [17]. However, when examining a new case before diagnostic biopsy, it is (usually) difficult merely by clinical or radiological examination to know whether a lesion has the potential to develop low or high malignancy or in some cases, is already malignant. In the following sections, we present an overview of studies that highlight possible events and mechanisms that may be associated with various complications using CNB.

### 2.2 Tissue trauma

Prior to CNB sampling, local anesthesia is used and a cut in the skin must be made. CNB uses larger hollow needles (~ 1.0–3.0 mm, outer diameter) compared with FNA (~ 0.4–0.9 mm). The sampling instrument is usually a biopsy gun with a spring‐loaded sampling device and ~ 3 up to 20 CNB samples may be taken in sequence during one sampling occasion, depending on the type, location and size of the lesion. One of the disadvantages of CNB, in addition to tissue trauma, is the difficulty in precise placement of the needle, which can be linked to the spring‐loaded device technology. With high needle velocity (5–10 m·s^−1^) several phenomena might occur. For instance, the temporary channel produced by cavitation will be significantly larger than the needle diameter and the kinetic energy induced is then absorbed by the tissues [18]. It is plausible that the total extent of tissue trauma is related to the deposited energy which is proportional to the number of shots, needle velocity, and needle diameter. The potential consequences of these ballistic effects are underestimated since current discussion about CNB focuses primarily on the needle diameter.

### 2.3 Cancer cell displacement and local seeding of cancer cells

Several studies have shown cancer cell displacement [19] and seeding of cells into adjacent tissues after CNB [20]. By comparison, needle tract seeding may be rare according to case reports. For example, after endoscopic ultrasound‐guided fine‐needle aspiration (EUS‐FNA) in pancreatic cancer (see Section 5.3), the general risk for complications was < 1%, and case reports on needle tract seeding were far less common [21]. Nevertheless, long‐term follow‐up after EUS‐FNA has been recommended to improve prognosis [22]. Interestingly, differences in seeding frequency were discussed in a meta study of 610 articles looking at tumor type in relation to diagnostic difficulties due to displacement of epithelial tumor cells [23]. Although the local seeding frequency was low within the needle tract, it was higher with CNB than with FNA. This result was confirmed in 2019 by Renshaw et al. [24] in a review of renal mass biopsies.

### 2.4 Tissue inflammation

Colotta et al. [25] suggested that cancer‐related inflammation of the TME—which is associated with downregulation of DNA repair pathways, increased proliferation, genetic instability, angiogenesis, metastasis and survival of malignant cells—should be recognized as a seventh hallmark of cancer. Later, using BC experimental mouse models, CNB‐induced inflammation was shown to be associated with an increase in metastasis. Mathenge et al. used a BALB/c model to monitor various immune cell populations and molecular markers after CNB and showed that myeloid‐derived suppressor cells (MDSCs) were elevated in the tumor tissue together with a > 3‐fold increase in circulating tumor cells (CTCs) and an enhanced frequency of lung metastasis [26]. Using the MMTV‐PyMT mouse model, Hobson et al. showed that a biopsy can stimulate cancer growth via angiogenesis and inflammation. An increase of pro‐inflammatory cytokines (*e.g*., IL‐6) was followed by an increase in lung metastasis [27]. Fu et al. [28] used the BALB/c model and found that CNB promoted lung metastasis. It was suggested that underlying mechanisms included TGF‐β1 mediated epithelial‐to‐mesenchymal transition (EMT). Numerous mechanisms may be involved in this CNB‐induced process, such as macrophage polarization, EMT, eosinophil recruitment and MDSCs [26, 29, 30].

Moreover, Chen et al. [31] showed recently that neutrophils may be indirectly involved in biopsy‐induced migration of glioma tumor cells in a mouse model (C57BL/6 with GL261cells) through the recruitment of macrophages to the TME. Zhao et al. [32] reviewed the intricate interplay between *chronic* inflammation and cancer development and suggested that consequences may be exaggerated tumor progression (via *e.g*. IL‐6) and development of treatment resistance, whereas *acute* inflammation may stimulate maturation of dendritic cells and anti‐tumor immune responses. If pro‐inflammatory factors persist during the acute inflammation phase, this may lead to chronic inflammatory conditions. Piotrowski et al. highlight in their review that surgical intervention elicits an acute inflammatory response which may impact the evolution of cancer cells [33]. In prostate cancer (PC) patients, transrectal biopsy is associated with an increased risk of infection including sepsis and patients are treated with prophylactic antibiotics, which in turn increases the risk for antimicrobial resistance [34, 35, 36]. To avoid the risk of infections, an alternative may be trans‐perineal prostate biopsy without antibiotic prophylaxis. Nevertheless, observations showed that a higher number of CNBs was associated with an increase in overall complications. In total, 6.0% of patients experienced various complications besides mild macrohematuria [37].

We conclude that infection and inflammation in the TME should be avoided and prevented. In experimental animal models as well as in several clinical studies, prophylactic administration of anti‐inflammatory drugs prior to biopsy or surgery showed significant reduction of metastases. Panigrahy et al. [38] showed in several models that preoperative administration of the anti‐inflammatory drug ketorolac prevents the formation of micro‐metastases and promotes long‐term survival. A large study based on data from 236.917 cancer patients who used non‐steroidal anti‐inflammatory drugs (NSAIDs) during the year prior to diagnosis indicated that advanced cancer was associated with an acute inflammatory process [39]. Interestingly, Pawitan et al. [40] also showed that the use of anti‐inflammatories was associated with tumor‐growth inhibition and that anti‐thrombotics (e.g., aspirin) was associated with a decrease of metastases.

A report by Alieva et al. [41] also supports the prevention of inflammation in conjunction with surgery or biopsy. Here, Alieva et al. showed that pretreating patients with dexamethasone can prevent biopsy‐induced inflammation and progression of tumors, possibly via inhibition of CCL2‐dependent recruitment and activation of macrophages.

Taken together, although some studies indicate that acute inflammation may be beneficial and contribute to anti‐tumor immune responses, several clinical and experimental studies show that prevention of inflammation in conjunction with biopsy may decrease the risk of cancer progression and metastasis.

### 2.5 Distant cancer cell dissemination

As mentioned above, Joosse et al. recently showed that prostate CNB may increase the release of CTCs into the blood circulation and shorten progression‐free survival. Although confirmatory trials with longer follow‐up are needed before changes in clinical routines can be implemented, these observations should be taken seriously and viewed in a wider context [9].

Given experiences from cytology diagnostics, it is likely that a dislodging of neoplastic cells by the biopsy procedure may be facilitated by the diminished cohesiveness of preferentially aneuploid cancer cells. Genetic alterations, aneuploidy, and chromosomal instability (CIN) of CTCs have been analyzed in several studies and reviewed extensively by Frietas et al. [42]. They conclude for instance that the spatial genetic heterogeneity of a tumor seems to be well reflected by the heterogeneity of CTCs from the same patient and that thyroid and pancreatic cancer patients with CTC chromosome 8 aneuploidy had worse overall survival than patients with near diploid CTCs. However, CTCs may have acquired mutations that are not identified in tissue biopsies, and may also have an increased intravasation competency, mobility, and drug resistance. Moreover, increased level of CIN in CTCs from patients with non‐small cell lung cancer (NSCLC) was related to increased risk of relapse. In fact, CIN studies of CTCs from several cancers appear to reflect spatial and temporal heterogeneity although additional biomarkers (BMs) are needed to effectively monitor disease progression via CTCs.

Recently, it was observed that *tumor cell debris* triggers release of pro‐inflammatory and proangiogenic cytokines (e.g., CCL2, IL‐6, and IL‐8) from macrophages and suggested that strategies for clearance of inflammation‐associated debris are needed to prevent debris‐stimulated metastasis and tumor growth [43]. While chemotherapy‐generated dead cell debris was in focus of this study, Haak et al. reviewed several pre‐clinical studies that also demonstrated that release of debris after surgery and biopsy can trigger tumor dormancy escape, stimulate inflammation, and enhance tumor angiogenesis, showing that these interventions may induce additional risks [44]. Interestingly, in 2014 we observed an increase in epithelial cellular material and debris in venous blood samples after diagnostic CNB of patients with prostate lesions [10].

The metastatic cascade is a complex multistep process. Pantel et al. reported that although the metastasis competent CTC subgroup represents a minimal part of the total number of CTCs, these metastasis competent CTCs can self‐replicate in a similar manner to stem cells. For instance, CTCs expressing EpCAM and ALDH1 (stem cell markers) could form metastases in immunodeficient mice. However, to fully understand metastatic progression, the interaction between CTCs and the TME must also be considered [45]. Analyses of CTCs have also demonstrated metastasis‐promoting tumor‐neutrophil interactions that may lead to survival and proliferation of CTCs. Moreover, CTC interactions with platelets, endothelial cells and cancer‐associated fibroblasts may also affect their metastatic capacity [46]. Interestingly, *in vitro* studies have shown that platelets react in different ways, depending on whether they interact with normal cells, tetraploid or aneuploid cancer cells [47].

In summary, it is therefore reasonable to assume that CNB sampling of tissue from aneuploid and more aggressive cancers results in CTCs with higher metastatic potential and consequently potentially increases the risk of distant metastases. Analysis of CTCs could be considered for evaluation of the safety of invasive diagnostic procedures such as CNB. However, despite all the studies discussed here, it is to date not clear *with absolute certainty* that the release of CTCs due to CNB causes relapse and/or poor survival [48]. A clinical trial with a focus on biopsy and surgery of BC and PC is ongoing (INJURMET) to further investigate these issues [https://cordis.europa.eu/project/id/834974].

## 3 Core needle biopsy versus fine‐needle aspiration biopsy

In general, the risk of complications is lower after FNA compared to CNB sampling. For instance, transrectal FNA sampling of the prostate was frequently used in the 1970s and 1980s and did not cause complications nor require prophylactic antibiotics [49]. In pancreatic cancer (PaC), preoperative endoscopic ultrasound‐guided FNA (EUS‐FNA) was not associated with increased risk of mortality [50]. These and numerous other examples have shown that the risk of complications associated with FNA sampling is very low.

Percutaneous transthoracic CNB is a method used to obtain diagnostic samples from lung lesions. The reported incidence of pneumothorax may range from 15% to 54%, but also pulmonary hemorrhage, hemothorax and chest wall hematoma which were among the commonly reported complications after percutaneous or transthoracic CNB [51]. Capalbo et al. [52] showed that transthoracic needle biopsy (TTNB) of lung lesions by FNA under CT guidance had a 2–3‐fold lower rate of complications and improved diagnostic accuracy compared to CNB. In addition, the average costs due to adverse events after FNA was reported as four‐fold lower than CNB based on TTNB sampling from 1700 patients [53]. A study of the cost‐effectiveness of the performance of FNA versus CNB in clinically palpable breast lesions, without considering possible complications, showed that CNB was almost 3 times more expensive [54]. A favorable cost‐effectiveness of FNA was also reported by Masood et al. [55].

In a study of biopsy methods in patients with BC, 990 patients were diagnosed using CNB and 1364 using FNA samples. CNB was associated with higher risk of mortality than FNA in the absence of post‐surgical radiotherapy [56].

We compared the rate of distant metastasis 5–15 years after diagnosing BC by FNA or CNB, using two matched cohorts and observed a significant increased rate of distant metastases in the CNB cohort. No difference, however, was observed in the rate of local metastases between the two groups [57].

Although several publications indicate an increased risk for patients undergoing CNB‐based compared to FNA‐based diagnostics, Dinarvand et al. from the MD Anderson Cancer Center (The University of Texas, US), reported that between 2009 and 2019, the number of breast lesion cases diagnosed by FNA decreased substantially (−43%). However, during the same period, the overall number of FNA‐based diagnostics for all types of lesions increased by 38% where the most pronounced increase consisted of EBUS‐FNA (162–757%) [58]. During the same period molecular testing and immunostaining of FNA samples increased 2.5‐ to 4.8‐fold. The gradual increase in CNB for BC diagnostics was explained by the need to discriminate *in situ* carcinomas from invasive cancers, better subclassification and a more standardized material for biomarker testing. This trend was supported by the European School of Oncology‐European Society for Medical Oncology consensus guidelines for advanced BC. The lack of standardization in cytology sample processing remains a concern for using predictive BMs. However, the International Academy of Cytology introduced the Yokohama System for breast FNA cytology in an effort to improve and standardize breast FNA reporting aiming at securing the validity of using FNA for breast lesions, and in the report describing this system, Hoda and Brachtel showed that the role of FNA would be a first test, ideally using the so‐called Rapid On‐Site Evaluation (ROSE), to triage those cases that may require more invasive and more expensive CNB. This procedure will increase sensitivity and specificity for FNA‐based diagnosis of benign and malignant cases [59]. By following the recommendations for FNA‐based diagnostics of breast lesions, given by the Yokohama System, high sensitivity and specificity of FNA for the assessment of malignancy can be achieved [60]. Efficient communication between surgical and medical oncologists, radiologists, and cytopathologists is the key to success for the diagnostic approach called the “triple test” (i.e. the combination of physical examination, imaging and FNA) [61]. This strategy has also been very useful for diagnostics of lung nodules [62]. If a study comparing CNB and FNA does not use a “triple test” including ROSE and/or if the cytopathologists has limited experience in FNA diagnostics, the result may be misleading.

In contrast to BC diagnostics, US‐guided FNA is widely used for the examination of thyroid nodules to prevent unnecessary surgery [63] and CT‐ and/or US‐guided FNA sampling from lung lesions has increased > 3‐fold from 2009 to 2019 [58].

## 4 Evolution of fine‐needle aspiration biopsy for cancer diagnostics and bibliometric analysis

**Fig. 1:**
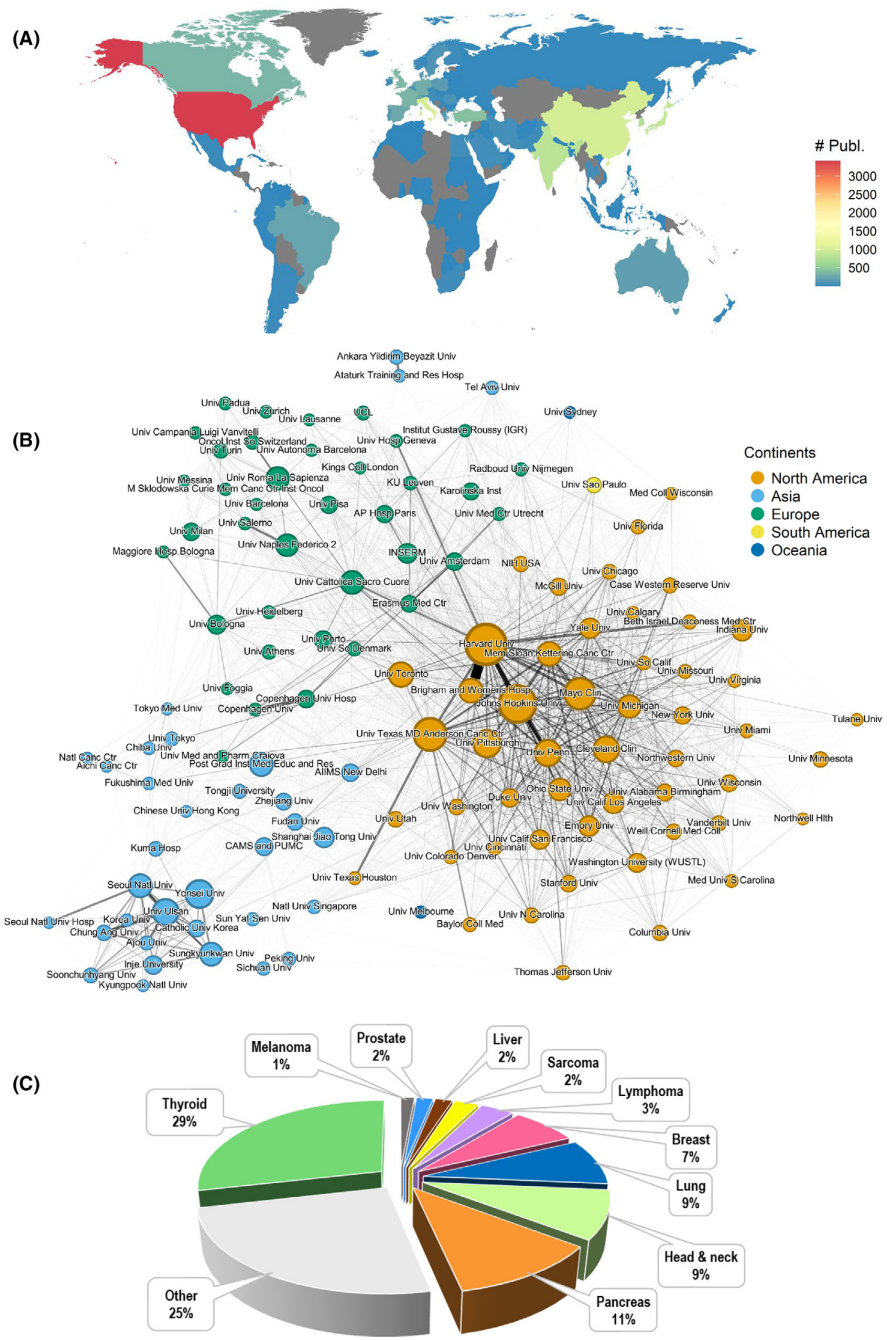
FNA in cancer diagnostics. Bibliometric analysis of 10 549 publications during the period 2012–2022. (A) Bibliometric analysis of the number of publications (#) with geolocations show predominant usage of FNA in North America, Europe, and East Asia. (B) Co‐publication network analysis show predominant research units with > 30 publications during the period. (C) Distribution of publications based on cancer types over the period (see Table 1).

The early development of FNA‐based cytology in cancer diagnostics has previously been reviewed extensively by others (*e.g*. [4]) FNA‐based cytology comprised ~ 10% of all publications in cancer cytopathology during the period 2012–2022 but cancer cytopathology in general is not within the scope of this review. Although the number of FNA publications reached a plateau in the 1990s due to a more frequent use of CNB, a more than double increase of the number of FNA publications occurred between 2000 and 2010. We conducted a bibliometric survey and co‐publication network analysis of “articles” and/or “reviews” on FNA‐based cancer diagnostics published between 2012 and 2022. The following search query was used to retrieve publications from PubMed: (((Biopsy, Fine‐Needle [MeSH Terms]) OR (Fine Needle Aspiration* OR Fine Needle Biopsy*)) AND ((neoplasms [MeSH Terms]) OR (neoplasm* or cancer* or tumor* or tumor*))). The publication set was restricted to publications indexed in both PubMed and Web of Science. This was done to use the address information from Web of Science, which is more structured than the address information in PubMed. The final set of publications consisted of 10 549 publications. To identify cancer types, we used the MeSH terms (including underlying terms) listed in Table 1. The Force Atlas 2 layout [64] was used for the co‐publishing network (Fig. 1B) and the network was restricted to organizations with at least 30 publications within the publication set.

**Table 1:** MeSH terms for identification of cancer types within the dataset. Fig. 1C shows the distribution of cancer types.

MeSH term(s)	Denotation and number of publications
“Thyroid Neoplasms”, “Thyroid Nodule”, “Thyroid Gland”	Thyroid_neoplasms (*N* = 3196)
“Pancreatic Neoplasms”	Pancreas_neoplasms (*N* = 1266)
“Head and Neck Neoplasms” (excl. “Thyroid Neoplasms”, “Thyroid Nodule”, “Thyroid Gland”)	Head_and_neck_neoplasms (*N* = 1001)
“Small Cell Lung Carcinoma”, “Carcinoma, Non‐Small‐Cell Lung”, “Lung Neoplasms”	Lung_neoplasms (*N* = 953)
“Breast”, “Breast Neoplasms”, “Carcinoma, Ductal, Breast”	Breast_neoplasms (*N* = 774)
“Liver Neoplasms”	Liver_neoplasms (*N* = 199)
“Prostatic Neoplasms”, “Prostatic Hyperplasia”	Prostatic_neoplasms (*N* = 195)
“Lymphoma”	Lymphoma (*N* = 391)
s“Sarcoma”	Sarcoma (*N* = 281)
“Melanoma”	Melanoma (*N* = 158)
“Neoplasms, Unknown Primary” (not shown in Fig. 1C, < 1%)	Neoplasms_metastasis_unknown (*N* = 34)

We note that the US dominates in the number of publications by a substantial margin, followed by East Asia (China, India, South Korea, Japan). Within Europe, Italy ranks highest in the number of publications (Fig. 1A). Figure 1B shows the affiliations that are most prevalent within each continent and how the network of co‐publications is distributed. Within the US, Harvard University (incl. Brigham and Women's Hospital linked to Harvard), Johns Hopkins University and MD Anderson Cancer Center are among the more prominent nodes. The figure also shows that research groups from Harvard and universities in the Netherlands have many joint publications. Figure 1C shows that published articles primarily including thyroid, but also pancreas, head & neck (except thyroid), and lung and breast, belong to the larger tumor groups where FNA has been used more frequently. Below we discuss the use of FNA within these tumor groups in more detail.

## 5 Molecular analysis of fine‐needle aspirates

Development of methodology for the analysis of molecular biomarkers (BMs) for clinical implementation in cancer diagnostics has accelerated rapidly in recent decades. These BMs can be of any type (DNA, RNA, protein), come from tissue samples or body fluids and be used for example, for early detection, diagnosis, malignancy grading, prediction of therapy response, decisions on change of therapeutics at different lines of treatment or monitoring in case of resistance development. Within PCM, it is becoming increasingly important to identify subgroups for clinical trials and even individuals who may be suitable for off‐label treatment [65].

In the absence of molecular BM analysis, FNA requires dedicated training of pathologists and radiologists to perform FNA sampling correctly and for cytopathologists to interpret the cytological smears, which takes many years of experience at the microscope. Even with the support of BM analysis, the sampling procedure including accurate precision placement of the needle, usually guided by imaging, still needs dedicated training. However, given that samples consist of representative cellular materials, a wide range of molecular analysis methods may be applied [66].

Interestingly, our bibliometric analysis between the years 2012–2022 showed an increase in the number of articles, on average 12% per year when we included molecular analyses of FNA samples by adding “AND (Pathology, Molecular [MeSH Terms]) OR (molecular)” to the previous search string. This subgroup has now reached ~ 20% of the total number of FNA publications. This complementary analysis indicates that there is a growing interest from the scientific community in the opportunities that the combination of FNA together with current molecular methods can offer. The report by Dinarvand et al. [58] confirms this observation. A frequently claimed disadvantage with FNA samples is the scarce amount of material for molecular testing. However, a “silent molecular revolution” is ongoing which was started about two decades ago by Pusztai et al. [67]. Using a median amount of two micrograms of total RNA per sample it was demonstrated that comprehensive transcription profiling can be performed on FNA biopsies to predict clinical ER (estrogen receptor) status and to measure conventional single‐gene prognostic markers such as ER and HER‐2 (human epidermal growth factor receptor 2). Since then, sensitivity has increased, and costs for RNA profiling have decreased substantially. Ullal et al. [68] performed targeted analysis in FNA samples of ~ 90 proteins using a DNA‐barcoded antibody‐based method. They showed that this highly sensitive and reproducible method may be developed as a clinical tool to predict drug response in lung cancer patients. Currently, we note that clinical routine requires just a few hundred nanograms DNA for whole genome sequencing and < 100 ng DNA for targeted mutation panels including 400–500 clinically relevant mutations [69, 70] [https://genohub.com/dna‐seq‐library‐preparation/]. Therefore, targeted mutation profiling of FNA material is now integrated into the daily routine at several molecular pathology laboratories. Here, FNA samples also offer an advantage over CNB in that the tumor cell fraction is significantly higher [5]. Currently, an awareness is slowly growing: In spite of the small sample size obtained by FNA, a wide range of molecular methods for prognostic and predictive BM testing can be applied, sometimes with better results than using histology materials [71, 72, 73]. We have recently published studies on molecular analysis of FNA samples using proximity extension assay, an ultrasensitive and highly specific and multiplex protein analysis method [74, 75, 76, 77]. Moreover, we show that using a simplified sample preparation method, the same preparation can be used for targeted analysis of RNAs and for mutation profiling. This development facilitates future implementation of a more advanced and multiomics‐based molecular cytology.

In addition, many of these techniques are very sensitive and can be used to analyze the minimal leftover material from the cytopathologist routine sampling procedure, allowing for a direct comparison between cyto‐morphology and molecular profiles of samples. These developments therefore pave the way for the use of molecular information to determine the presence of established drug targets or targets for pharmaceuticals under development. It will most likely also be possible to characterize the immune phenotype at the molecular level to support immunotherapy decisions. Below, we describe the state‐of‐the‐art of molecular cytology in more detail, within some tumor groups.

### 5.1 Thyroid cancer diagnostics by FNA

Thyroid nodules are frequently detected in the population and a majority of these are benign. To reduce unnecessary surgery of benign lesions, a patient‐friendly FNA biopsy is a cost‐effective and well‐established method to obtain cellular material for examination of thyroid nodules [78, 79]. However, the diagnostic accuracy varies between histological subtypes and in up to about one third of suspected malignancies, additional molecular tests may be needed [80]. Molecular markers such as BRAF, RAS and RET/PTC mutations or ICC (immunocytochemistry) markers (e.g., Galectin‐3, CD44) may be used to support diagnostics. TERT promotor mutation is associated to poor prognosis and analysis by digital droplet PCR showed that this method may guide surgical decisions in a subset of patients [81].

RNA expression‐based classifiers have also been tested to provide decision support in cytologically indeterminant thyroid nodules (“suspicion of a follicular neoplasm or follicular neoplasm”) with promising results [82].

However, this type of commercial test needs extensive validation before clinical implementation [83]. In the future, multi‐omics approaches may well be of value for early diagnosis of, for example, potentially metastatic papillary thyroid cancer (PTC) but this will require analysis of larger cohorts for training of models and external validation [84].

### 5.2 Lung cancer diagnostics by FNA

For cytopathological evaluation of lung samples, obtaining representative and enough material for diagnostics is crucial. Previously, sputum was the only sample type with relatively well‐defined criteria, but now there are published definitions of what constitutes a sufficient sample and in the case of CT‐guided transthoracic FNA, ROSE is used for assessment of material quantity and representativeness [85]. In NSCLC, a limited ICC panel (TTF1, Napsin A) is used to differentiate between adenocarcinoma and squamous cell carcinoma. Moreover, FNA smears or cell block material may be used for ICC analysis of PD‐L1 protein levels [86]. Here, cell blocks could be used more frequently [87], however, analysis of smears might be the only option when samples are scarce. Residual material from the FNA needle can be used for mutation profiling by targeted NGS panels to identify actionable mutations or fusion genes, not only in NSCLC [88]. Many of the above‐mentioned methods are currently implemented in clinical practice and we have shown that in addition to these methods, we can use residual materials for targeted protein profiling [76]. In addition, RNA‐based NGS for gene fusion analysis such as EML4‐ALK and EZR‐ROS1, is increasingly successful in cytology and has reached a success rate of 92% in NSCLC. Ramani et al. concluded here that cytology material is an excellent source for predictive NGS fusion BM testing [89]. Taken together, we find numerous studies that convincingly show the value of comprehensive molecular cytological analyses in lung cancer, including practical advice for quality control and correct handling of samples [90].

### 5.3 Pancreatic cancer diagnostics by FNA

Management of patients with pancreatic cancer (PaC) is very challenging and there are not yet any targetable molecules in clinical practice for PCM. Effective diagnostic tools for PaC are endoscopic ultrasound‐guided fine‐needle aspiration (EUS‐FNA) or biopsy (EUS‐FNB) [91]. Although EUS‐FNA had lower accuracy in this study, the risk for complications is very low and manageable given that recommendations are followed [50, 92, 93]. Molecular testing of FNA materials from PaC is increasing and may be used for example, for NGS based mutation profiling [94, 95, 96]. KRAS mutations are frequent in PaC and targeting KRAS‐G12Cmay be a promising way forward, and several clinical trials are ongoing in parallel with other actionable molecular targets using drugs and antibody alone or in combination [97]. In practice, however, the G12D mutation is the most common in pancreatic cancer and present in approximately 35% of people diagnosed with the disease. This mutation may be targeted by an experimental drug, MRTX1133. Thus, relevant KRAS mutations in PaC may be identified using FNA. A large fraction of PaC is aneuploid and highly malignant, therefore FNA sampling from PaC may be of value to further reduce the risk of complications since this method is less invasive compared to FNB.

### 5.4 Breast cancer diagnostics by FNA

Historically, FNA has been very useful in BC diagnostics. For instance, in one study based on 2594 patients with non‐palpable lesions it was concluded that the combination of mammography and stereotactic FNA sampling (precision placement of the needle) showed high sensitivity for early diagnosis of BC [98]. Only few studies compare long‐term effects of FNA versus CNB. Kim et al. [99] did not find any significant differences in overall survival between BC patients diagnosed with these methods. However, as mentioned above, we observed a significantly higher rate of distant metastasis in BC patients diagnosed by CNB versus FNA [57]. Although there has been a general decline in the use of FNA for BC diagnostics since 1990s, Nassar et al. claim that FNA has several important advantages compared with CNB, in particular for metastatic lesions and when combined with molecular analysis [100]. Using the “triple test” and following established guidelines as mentioned above, FNA‐based BC diagnostics is very useful [60]. Foukakis et al. [101] analyzed FNA samples with gene expression profiling from locally advanced inoperable or metastatic BC in a multicenter, randomized phase III trial. They observed that response to chemotherapy was associated with an immune module score in patients with ER‐positive or luminal tumors.

Delaloge et al. describe a model for rapid BC diagnostics within a “one‐stop breast clinic” providing high diagnostic accuracy and favorable cost‐effectiveness. During 2004 to 2012, > 10 000 patients with suspected BC were examined with US‐guided FNA and in 75% of cases a final diagnosis was obtained on the same day as the FNA sampling was performed [102, 103]. In addition, Chen et al. recently showed high sensitivity and specificity for initial diagnostics of breast lesions. It was suggested that FNA could be considered as an initial diagnostic tool as well as for triaging patients [104]. However, our bibliometric analysis and studies by others showed a declining trend for FNA‐based BC diagnostics, as previously mentioned [58]. This is unfortunate, given an increased awareness of possible complications associated with CNB sampling in cases of more aggressive BC and the rich molecular information that may be obtained by minimal invasive FNA [75].

### 5.5 Prostate cancer diagnostics by FNA

FNA‐based prostate cancer (PC) diagnostics was used extensively for several decades (1960s–1980s) with minimal complications [49, 105]. However, FNA was gradually replaced by CNB although the diagnostic accuracy was similar [106]. The Gleason scoring system was gradually introduced during the 1990s and required CNB sampling although FNA was still used rather frequently in 2011 in Sweden [107, 108]. A minority of PC is of highly malignant subtype and in these cases CNB sampling may be associated with unnecessary risks for the patient, as it is with CNB sampling of highly malignant and aneuploid BC [109]. Currently, BMs in liquid biopsy together with MRI may be used to identify patients at risk for PC and thereby lower the number of unnecessary CNB samples [110]. We may, however, experience an increased use of image‐guided FNA‐based diagnostics as a complement to these methods, especially for “unclear” MRI sites [77, 111]. Here, we may also monitor immune targets for advanced PC. Only if these new FNA‐based diagnostics are inconclusive, would CNB sampling be an option, thereby lowering the frequency of unnecessary CNB sampling.

### 5.6 Head & neck cancer diagnostics by FNA

FNA combined with US may be used for first‐line evaluation of head and neck lesions. After exclusion of patients with lymph nodes, frequently diagnosed as metastasis or lymphomas, and thyroid lesions (see above), patients with salivary gland nodules represent a major group [112]. Salivary gland cancers (SGC) consist of many different subtypes of which a third are more malignant and the remaining fraction are of low to intermediate grade. Some of these cancers may transform into high‐grade variants. In many cases, FNA may be useful for detailed characterization and patient treatment management [113]. Recently, Oh et al. [114] showed that a prediction model based on a 29‐plex BM panel analyzed by a novel and rapid technique, could be used to distinguish FNA samples from patients with benign salivary gland lesions from SGC with 88% accuracy.

To build confidence in FNA‐based BM ICC testing, benchmarking versus corresponding IHC BMs are valuable. Liu et al. [115] analyzed PD‐L1 in 21 patients with head and neck squamous cell carcinoma (HNSCC) and found a good consistency between paired histology and cytology‐based assessments.

This consistency between histology and cytology at a broader molecular level has also been confirmed by analysis of several other tumor types, for instance, Dupain et al. [116] analyzed more than 100 molecular alterations (mutations, amplifications, deletions, SNVs, etc.) in various tumors from 61 patients and 80% of the alterations were concordant between FNA and CNB samples.

### 5.7 Sarcoma diagnostics by FNA

Sarcomas are diverse non‐epithelial malignancies of bone or soft tissue origin with more than 70 subtypes where FNA may provide conclusive diagnostics [117]. In addition, due to a high risk of recurrence, low risk FNA sampling of sarcoma cells may be considered before CNB. Molecular markers such as the fusion gene SYT‐SSX may be useful in the diagnosis of synovial sarcomas [118]. Moreover, in analogy to thyroid cancers, TERT promotor is overexpressed in more aggressive chondrosarcomas, and has the potential as prognostic marker that could be decisive for telomerase‐targeted therapy [119].

## 6 Monitoring of molecular dynamics during treatment by longitudinal FNA sampling

Repeated FNA sampling to monitor molecular dynamics in tissues during therapy has a huge potential. For instance, longitudinal FNA samples have been collected and analyzed by single cell RNA sequencing from patients with chronic hepatitis B to monitor the effect of antiviral therapy [120]. Results showed an increased liver resident CD8^+^ T cell population at baseline and an evolving activated immune signature during liver damage.

Repeated sampling may also be valuable in scenarios where patients have low malignant or benign lesions but would most likely be highly desirable during therapy of patients with advanced cancers where other sampling methods cannot be used. In the first case, repeated FNA sampling of, for example, thyroid lesions classified as “atypia of undetermined significance/follicular lesion of undetermined significance” (Bethesda class III) can reclassify a majority of these lesions as benign and thereby avoid surgery/diagnostic lobectomy [121]. In the latter scenario, patients with advanced, inoperable metastatic cancers frequently develop relapses. For instance, 30–55% of patients with surgically removed NSCLC relapse, and 50–70% of ovarian carcinomas recur within 1 year after surgery combined with chemotherapy, [122]. Numerous intrinsic and acquired drug resistance mechanisms may evolve and molecular characterization strategies including sequencing, transcriptomics, immunophenotyping, and epigenetic profiling are then needed for clinical decision making [123, 124]. Here, repeated, serial FNA sampling may then offer unique opportunities for longitudinal assessment of the molecular dynamics within the tumor microenvironment (TME) [2]. We have observed increased levels of many intercellular proteins in HER‐2 positive and triple negative versus luminal A BCs in FNA samples indicating that the molecular dynamics in the TME may be monitored [75]. Using gene expression profiling, Chen et al. [125] reported that adaptive immune signatures in baseline melanoma biopsies were predictive of response to immune checkpoint blockade (ICB) and longitudinal sampling combined with molecular analysis revealed mechanisms of resistance. In FNA samples from NSCLC patients, we identified several ICB resistance‐associated proteins (*e.g*., CCL3, CCL4, CCL23, CXCL5, IL6, IL8, GZMA, GZMB) [76] previously identified by Chen et al. [125]. Oh et al. [114] developed an antibody‐based method for serial immune profiling of the TME by FNA sampling. Samples were obtained from mouse tumors treated with anti‐PD‐1, and higher levels of IL‐12 in dendritic cells were observed in responders as compared to non‐responders. Moreover, in one patient, serial time course analysis of the TME in HNSCC during immunotherapy showed a decrease in the macrophage fraction and an increase in NK cell, CD8^+^ T cell, and dendritic cell fractions. Interestingly, benchmarking versus flow cytometry‐based analysis of the same protein markers showed high correlation.

In a case study, samples were collected longitudinally by EUS‐guided FNA in two patients with inoperable PaC for analysis of changes in the gene expression profile associated with radiofrequency ablation (RFA). Among several interesting changes in gene expression, a > 2‐fold increase in immune checkpoint genes was observed within the TME after RFA. It was concluded that this combined technique has a significant potential for increased understanding of the molecular dynamics within the TME during therapy and may be useful in clinical trials [126].

Iacono et al. analyzed EGFR‐T790M mutations and MET amplifications in biopsies (e.g., FNA) in 27 NSCLC patients pre‐ and post‐EGFR therapy [127]. This study identified both T790M losses and gains in longitudinal biopsies and demonstrates the need to analyze both spatial and temporal molecular heterogeneity during therapy.

These examples of longitudinal studies suggest that molecular alterations in the TME, including analysis of actionable targets may be characterized by FNA sampling and should be used more frequently in clinical trials to replace the more invasive CNB sampling.

## 7 Concluding remarks and future perspectives

We have, in this review, described a rapid development in cancer diagnostics over the last decade which opens opportunities for a new emerging translational research area, “Integrative Molecular Cytology” that focuses on the *integration* of information from rich molecular data (mutations, gene‐ and protein expression) and morphology (digital cytology) in support of improved diagnostics, optimal therapy decisions and an overall improved PCM strategy. This development has also been recognized by several others [5, 66]. Thanks to the combination of image‐guided and minimally invasive FNA sampling, repeated and longitudinal sampling allows for molecular monitoring of tumor heterogeneity and treatment responses for each individual patient. Considering the perspective of the possible complications associated with the a more traumatic biopsy method, it is reasonable to suggest that CNB could be replaced by FNA, particularly for the monitoring of therapy responses, but also for early diagnosis and analysis of metastases [128]. While many potential biomarker signatures and recently developed molecular methods need clinical validation before implementation, we nevertheless see a huge untapped potential for future molecular cytology.

In this review, we have not discussed how advances in artificial intelligence (AI) may support the development of molecular cytology. However, we and others are convinced that various AI tools will provide strong support and potentiate the opportunities for an integrated analysis of complex data [129, 130].

Finally, we conclude that Integrative Molecular Cytology has a substantial potential to increase the precision of diagnostics and similarly the individualization of treatment decisions, contribute to guide clinical trials, and to the implementation of PCM in general, to improve patient quality of life soon.

## Author contributions

GA conceived and designed the project. GA and BF were responsible for literature search, review of the literature and the original draft of the manuscript. BF complied data and was responsible for administration, tables, and figs RL was responsible for acquisition of the financial support, coordination of the work, review, and final edition of the manuscript.

## Conflict of interest

The authors declare no conflict of interest.
